# Synthesis of Methyldopa–Tin Complexes and Their Applicability as Photostabilizers for the Protection of Polyvinyl Chloride against Photolysis

**DOI:** 10.3390/polym14214590

**Published:** 2022-10-28

**Authors:** Noor Naoom, Emad Yousif, Dina S. Ahmed, Benson M. Kariuki, Gamal A. El-Hiti

**Affiliations:** 1 Department of Chemistry, College of Science, Al-Nahrain University, Baghdad 64021, Iraq; 2 Department of Medical Instrumentation Engineering, Al-Mansour University College, Baghdad 64201, Iraq; 3 School of Chemistry, Cardiff University, Main Building, Park Place, Cardiff CF10 3AT, UK; 4 Department of Optometry, College of Applied Medical Sciences, King Saud University, Riyadh 11433, Saudi Arabia

**Keywords:** methyldopa–tin complexes, photolysis, polyvinyl chloride, weight loss, molecular weight depression, surface morphology

## Abstract

Polyvinyl chloride (PVC) is a ubiquitous thermoplastic that is produced on an enormous industrial scale to meet growing global demand. PVC has many favorable properties and is used in various applications. However, photodecomposition occurs when harsh conditions, such as high temperatures in the presence of oxygen and moisture, are encountered. Thus, PVC is blended with additives to increase its resistance to deterioration caused by exposure to ultraviolet light. In the current research, five methyldopa–tin complexes were synthesized and characterized. The methyldopa–tin complexes were mixed with PVC at a concentration of 0.5% by weight, and thin films were produced. The capability of the complexes to protect PVC from irradiation was shown by a reduction in the formation of small residues containing alcohols, ketones, and alkenes, as well as in weight loss and in the molecular weight of irradiated polymeric blends. In addition, the use of the new additives significantly reduced the roughness factor of the irradiated films. The additives containing aromatic substituents (phenyl rings) were more effective compared to those comprising aliphatic substituents (butyl and methyl groups). Methyldopa–tin complexes have the ability to absorb radiation, coordinate with polymeric chains, and act as radical, peroxide, and hydrogen chloride scavengers.

## 1. Introduction

The demand for plastics is projected to keep increasing due to their versatile properties [1]. Plastics are strong, light, and inexpensive and can be produced in different forms to suit specific applications [2,3,4]. Plastics are, therefore, efficient replacements for some common construction materials, such as steel, wood, and glass. However, a drawback is that plastics suffer from photodegradation when exposed to ultraviolet (UV) light at high temperatures in environments rich in oxygen [5]. Photodegradation leads to deterioration in their mechanical and physical properties resulting in color changes, deformation, and the appearance of cracks [6]. It is, therefore, necessary to enhance the long-term resistance of plastics to photodegradation in hot and humid conditions during manufacturing [7].

Polyvinyl chloride (PVC) is a thermoplastic polymer that ranks in the top three, along with polyethylene and polypropylene, in terms of commercial production [8,9]. PVC is inexpensive, possesses excellent mechanical properties, is available in different forms, and is simple to process and shape. It has many industrial applications, including water piping, insulation, flooring, roofing, packaging, medical instruments, cars, and furniture [10,11]. The downside, however, is that PVC waste is hazardous to humans, animals, and the environment. Furthermore, the photodecomposition of the material can occur in the presence of oxygen at high temperatures if PVC is exposed to light in humid conditions [12,13]. Two strategies are, therefore, employed to reduce PVC waste generation and its photodecomposition. The first strategy involves recycling and reusing PVC waste. Various effective processes have been employed for the recycling of PVC [14,15,16]. The second strategy involves the addition of stabilizers to suppress the photodecomposition, which can lead to the deterioration of properties and shortening of the duration of useful life for PVC [17]. PVC photodegradation is autocatalytic and leads to the production of chains that contain conjugated double bonds as a result of dehydrochlorination and the formation of volatile products. In addition, it causes a decrease in the molecular weight, a reduction in weight, and discoloration of PVC. The main reasons for PVC photodegradation are chain scissions and cross-linking due to the elimination of hydrogen chloride (HCl) [18,19,20,21,22].

Recent research has focused on synthesizing new organic PVC stabilizers with high aromaticity and heteroatom concentrations [5]. These additives act to protect polymeric materials from harmful irradiation and reduce the changes in mechanical properties, cracking, and discoloration [23,24]. Photostabilizers absorb energy from irradiation and quench active and volatile species, such as radicals and HCl [25]. The additives should be nonvolatile, nontoxic, and inexpensive and used at low concentrations to avoid changes in the color of PVC [26]. Many potentially commercial PVC stabilizers are banned mainly because of associated hazards or the requirement for co-stabilizers [27,28,29,30]. Alternatives such as Schiff bases, polyphosphates, and complexes containing tin and aromatic moieties were synthesized, and their role as PVC additives has been assessed [31,32,33,34,35].

Tin complexes containing organic residues are highly stable and have various medicinal applications [36,37,38,39,40]. Additionally, tin complexes are used in catalysis, wood preservatives, agrochemicals, disinfectants, biocides, and polymer additives [41]. Methyldopa is an antihypertensive medication and can be used to lower blood pressure [42]. It is inexpensive, commercially available, stable, nontoxic, and contains an aromatic moiety and heteroatoms (36.9% by weight). Therefore, methyldopa is a good candidate for inclusion in the additives used to protect PVC from photolysis. We report the synthesis of new methyldopa–tin complexes and investigate their effect on PVC photodecomposition.

## 2. Materials and Methods

### 2.1. Materials and Samples Preparation

Merck (Gillingham, UK) supplied tin chlorides (95–98%), methyldopa (99%), and solvents (analytical grades), which were used as received. The PVC (M_V_ = ca. 180,000) was purchased from Petkim Petrokimya (Istanbul, Turkey). The PVC blends were prepared in a Kerry PUL 55 ultrasonic bath (Kerry Ultrasonics Ltd., Hitchin, UK). An accelerated weather meter QUV tester (Homestead, FL, USA) was used for the irradiation (365 nm; 6.2 × 10^−9^ Einstein dm^−3^ s^−1^) of the films at room temperature. The tester is equipped with two identical 40 watts fluorescent lamps (UV-B 365), one on each side. The distance between the films and the tester was kept at 10 cm. The PVC materials were rotated occasionally in order for the films to be irradiated equally from all sides.

### 2.2. Methods

The microanalytical analysis was performed using a Shimadzu AA-6880 spectrophotometer (Tokyo, Japan). A Shimadzu FT-IR8400S (Tokyo, Japan) was used to record the infrared (IR) spectra (400–4000 cm^−1^). The nuclear magnetic resonance (NMR) spectra were recorded in deuterated dimethyl sulfoxide (DMSO-d_6_) using a Bruker Advance DRX 400 MHz spectrometer (Zürich, Switzerland). An Ostwald U-Tube Viscometer (Ambala, India) was used to measure the viscosity of solutions. A Bruker XFlash 6-10 (Tokyo, Japan) was used to record energy-dispersive X-ray (EDX) spectra. Before the EDX spectra were recorded, the films were coated with a thin layer (ca. 15 mm) of gold (Au). The optical images were recorded using a Meiji Techno microscope (Tokyo, Japan). The atomic force microscopy (AFM) images were captured on a Veeco instrument (Plainview, NY, USA). The scanning electron microscopy (SEM) images were recorded using an Inspect S50 microscope (FEI Company, Czechia, Czech Republic; 15 kV).

### 2.3. Synthesis of **1** and **2**

**Scheme 1:**
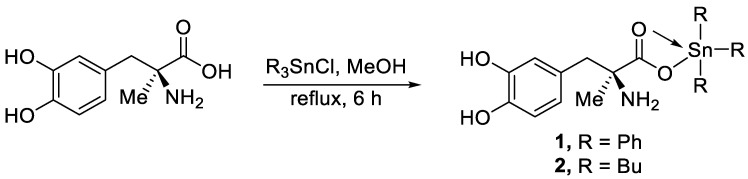
Synthesis of **1** and **2**.

A mixture of methyldopa (0.21 g, 1 mmol) and Ph_3_SnCl (0.39 g, 1 mmol) or Bu_3_SnCl (0.33 g, 1 mmol) in MeOH (30 mL) was refluxed for 6 h (Scheme 1). The white solid obtained was filtered, washed with MeOH, and dried in a vacuum oven at 45 °C for 6 h to give **1** or **2** in a high yield (Table 1).

### 2.4. Synthesis of **3**–**5**

**Scheme 2:**
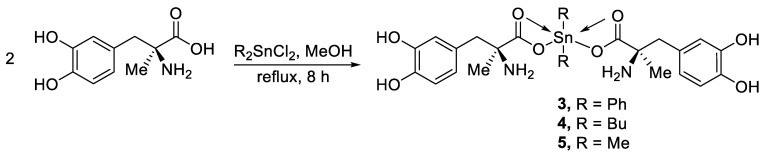
Synthesis of **3–5**.

A mixture of methyldopa (0.42 g, 2 mmol) and Ph_2_SnCl_2_ (0.34 g, 1 mmol), Bu_2_SnCl_2_ (0.30 g, 1 mmol), or Me_2_SnCl_2_ (0.22 g, 1 mmol) in MeOH (30 mL) was refluxed for 8 h (Scheme 2). The resulting white solid was filtered, washed with MeOH, and dried in a vacuum oven at 45 °C for 6 h to produce **3**, **4**, or **5** in a high yield (Table 1).

### 2.5. Preparation of PVC Films

Tetrahydrofuran (THF; 100 mL) was added to a mixture of the appropriate complex (25 mg) and PVC (5.0 g). The mixture was stirred for 2 h at 25 °C to ensure the formation of a homogenous blend. The solution was poured into a glass plate containing holes (thickness = 40 μm). The plate was left in the air at 25 °C for 24 h to dry. A vacuum oven (40 °C; 8 h) was used to ensure the dryness of the films.

### 2.6. Assessment of IR Spectra

The PVC photodecomposition process generates active species (e.g., free radicals), which initiates the elimination of volatiles and bond cleavage [43]. As a result, fragments containing hydroxyl (OH; e.g., alcohols), carbonyl (C=O; e.g., ketones), and carbon−carbon double bonds (C=C; e.g., alkenes) are produced [44,45]. The IR absorption bands corresponding to the OH, C=O, and C=C groups appear at about 3497, 1714, and 1612 cm^−1^, respectively. The intensities of the peaks corresponding to these functional groups can be monitored using IR spectroscopy during irradiation. A standard peak that appeared at 1328 cm^−1^ (C–H bond) was chosen for comparison [46]. Equation (1) was used to calculate the indices (I_s_) of the hydroxyl, carbonyl, and polyene groups (I_OH_, I_C=O_, and I_C=C_, respectively) as a function of the absorbances of the functional groups (A_s_) and the standard peak (A_r_).

(1) $$I_{s}=\frac{A_{s}}{A_{r}}$$

### 2.7. Assessment of Weight Loss

PVC irradiation causes weight loss due to the formation of small fragments. Equation (2) can be used to calculate the percentage of PVC weight loss at a time (t) of irradiation [47]. The weight loss was calculated using the weights of the non-irradiated and irradiated PVC films (W_0_ and W_t_, respectively) [47].

(2) $$\mathrm{Weight}\;\mathrm{loss}\;\left(\%\right)=\frac{W_{0}-W_{t}}{W_{0}}\times 100$$

### 2.8. Assessment of Average Molecular Weight (M_V_)

PVC irradiation decreases the Mv as a result of the elimination of small residues, including volatiles. Following irradiation, the blends were dissolved in a solvent (THF), and their intrinsic viscosities [η] were measured. Filtration was necessary to remove any insoluble polymeric residues as a result of cross-linking of PVC. The decreases in Mv are directly proportional to the [η]. The Mark–Houwink relationship (Equation (3)) was used to calculate the Mv of the irradiated blends [48]. The viscosity of the PVC films was determined in THF at 25 °C.

(3) $$\left[\eta \right]=1.38\times 10^{-4}{\;M}_{v}^{0.77}$$

## 3. Results and Discussion

### 3.1. Synthesis of Complexes **1**–**5**

The reaction of methyldopa and trisubstituted tin chlorides in a 1:1 molar proportion gave complexes **1** and **2** (Scheme 1) 83% and 74% yields, respectively (Table 1). The reaction of methyldopa with disubstituted tin dichlorides in a 2:1 molar proportion gave complexes **3–5** (Scheme 2) in 78–84% yields (Table 1). The procedures were simple, effective, general, repeatable, and high yielding. In addition, the introduction of substituents (aliphatic and aromatic) in different numbers (di and tri) to the skeleton of the complexes was achievable.

The IR spectra of methyldopa–tin complexes **1–5** showed several absorption bands corresponding to the OH (3217–3279 cm^−1^), Sn–C (511–530 cm^−1^), and Sn–O (443–457 cm^−1^) bonds (Table 2). The presence of two absorption bands (symmetric and anti-symmetric) for the NH_2_ (3473–3409 cm^−1^) was observed in most cases (Table 2). The vibration signal of the asymmetric carbonyl group has shifted to a higher frequency due to coordination between the tin atom and the carboxylate group (COO^−^) oxygen. The difference (Δv) between the asymmetric (asym) and symmetric (sym) vibration frequencies of the COO^−^ group was calculated to be between 237 and 285 cm^−1^. A value of Δv in this range indicates an intermediate state between monodentate and bidentate (anisobidentate) asymmetry [49,50].

The ^1^H NMR spectra confirmed the presence of protons from both methyldopa and substituted tin chloride (Table 3). It was noted that the peaks corresponding to the OH and NH_2_ protons were broad, possibly due to the partial proton exchange with the deuterated solvent (DMSO-d_6_).

The structures of **1–5** were established further by the ^13^C NMR spectral data (Table 4). The carbon atoms of the carbonyl groups were detected at very low fields in the region of 172.9–174.2 ppm.

### 3.2. The EDX Spectroscopy of PVC Films

**Figure 1:**
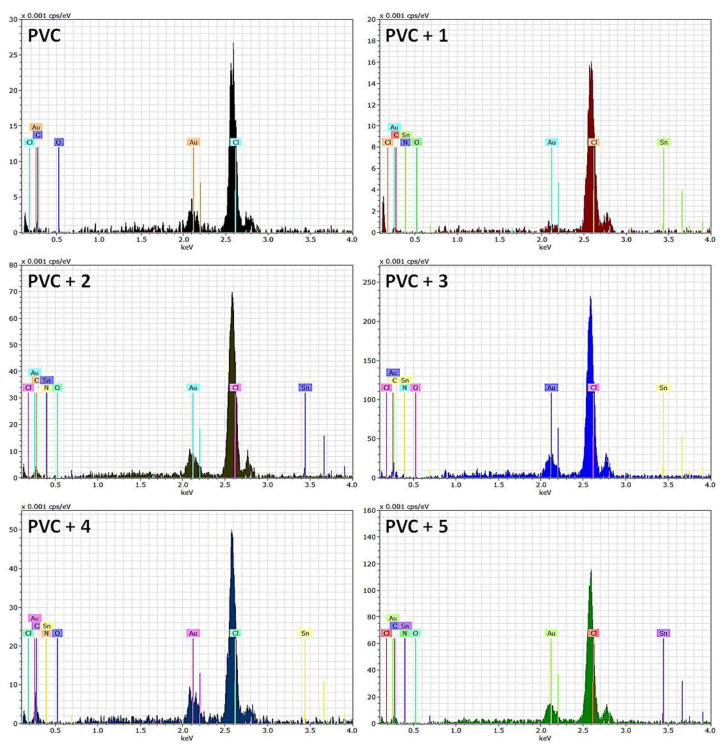
EDX spectra of PVC films.

Homogenous, colorless thin films were produced by mixing the appropriate methyldopa–tin complex (0.5% by weight) with PVC. EDX spectroscopy was used to evaluate the elemental composition of the PVC blends [51,52,53]. The EDX spectra of the non-irradiated films exhibited highly abundant absorption bands corresponding to the Cl atoms of PVC, as well as those for N and O from methyldopa and Sn, but in lower proportions (Figure 1). The peak assignment agrees with those previously reported for PVC blends with other additives containing tin and related elements [31].

The analysis of the EDX mapping indicated that metal complexes were completely dispersed and compatible with the polymeric matrix. The elements from metal complexes (Sn, N, and O) were distributed homogenously in a similar percentage. Following irradiation, the chlorine content dropped due to the elimination of volatiles, including HCl. The blends containing methyldopa–tin complex showed a lesser decrease in the chlorine content compared to the blank film. Clearly, additives **1–5** can be used to reduce PVC photodecomposition. The irradiated PVC film containing complex **1** shows the highest chlorine content.

**Figure 2:**
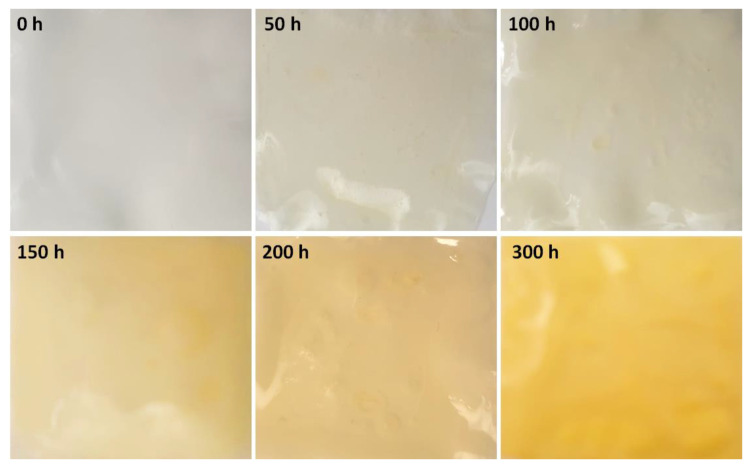
Color of PVC films after different irradiation times.

It was noted that the color of the PVC films became darker as the irradiation time increased (Figure 2).

### 3.3. IR Spectral Study of PVC Films

**Figure 3:**
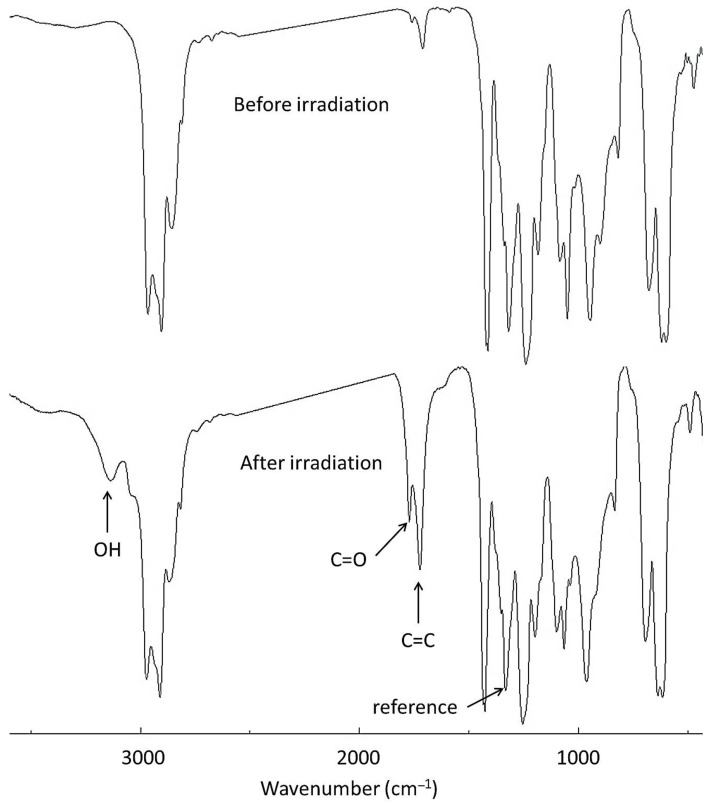
FTIR spectrum of PVC film before and after irradiation (300 h).

Photooxidation of PVC causes the degradation of polymeric chains and produces fragments containing alcohols (OH), ketones (C=O), and polyenes (C=C) [54,55]. The IR spectra of the PVC films were recorded at intervals of 50 h during irradiation. The intensity of the absorption peaks corresponding to the OH (3497 cm^−1^), C=O (1714 cm^−1^), and C=C (1612 cm^−1^) increased due to irradiation (Figure 3). Therefore, the intensities of these peaks were monitored over time and compared to the C–H bond (1328 cm^−1^), which act as a reference. Irradiation has little effect on the intensity of the C–H bonds since they are highly stable.

**Figure 4:**
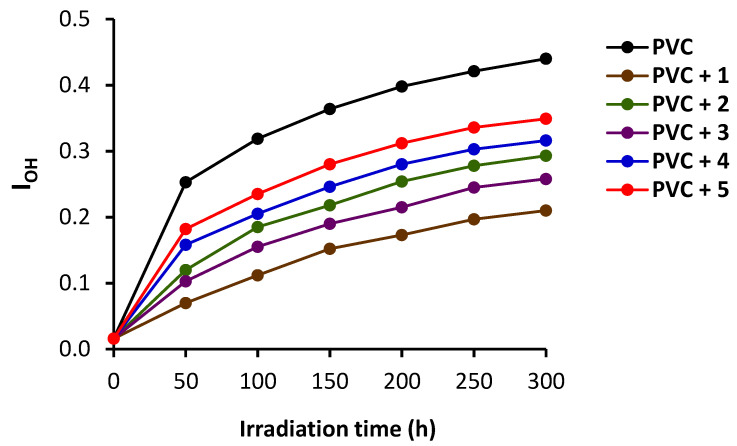
The I_OH_ of the irradiated PVC films.

**Figure 5:**
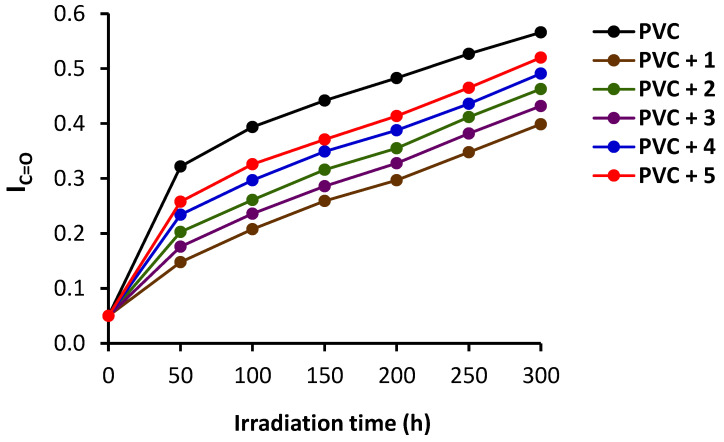
The I_C=O_ of the irradiated PVC films.

**Figure 6:**
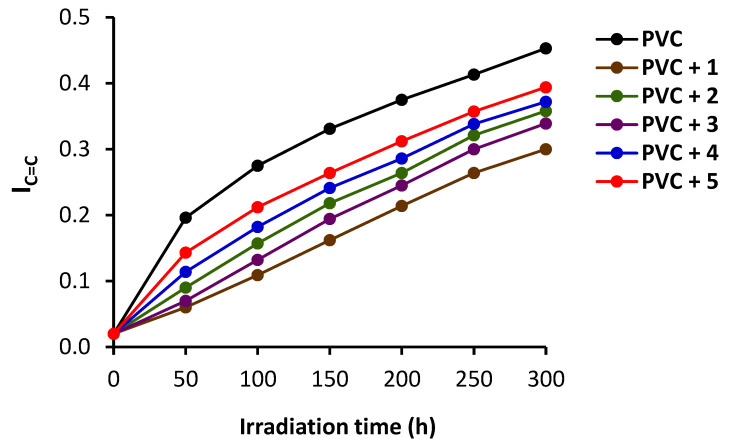
The I_C=C_ of the irradiated PVC films.

The indices of the OH (I_OH_), C=O (I_C=O_), and C=C (I_C=C_) groups of the irradiated films were calculated using Equation (1) and presented graphically in Figure 4, Figure 5 and Figure 6, respectively. The I_OH_, I_C=O_, and I_C=C_ increased significantly as the irradiation process progressed. The increases were very sharp at the beginning of the irradiation and then continued to increase steadily. The films containing **1–5** showed lower increases in the indices of functional groups compared to pure PVC film. It was clear that methyldopa–tin complexes act as photostabilizers to protect PVC. The additives with the highest aromaticity content (i.e., **1** and **3**) showed the greatest stabilizing effect. The complexes were stabilized in the following order: triphenyl (**1**), diphenyl (**3**), tributyl (**2**), dibutyl (**4**), and dimethyl (**5**). The complexes interact with the active species (e.g., radicals), causing PVC photodegradation and the formation of active intermediates. The stability of the intermediates is highly dependent on the number and type of aromatic moieties present. Aromatic rings tend to reduce the energy of intermediates through resonance. At the end of the irradiation process, the I_OH_ were 0.44 (blank PVC film), 0.21 (PVC + **1**), 0.29 (PVC + **2**), 0.26 (PVC + **3**), 0.32 (PVC + **4**), and 0.35 (PVC + **5**). Similar trends were observed for the I_C=O_ and I_C=C_ values.

### 3.4. Weight Loss Investigation of PVC Films

**Figure 7:**
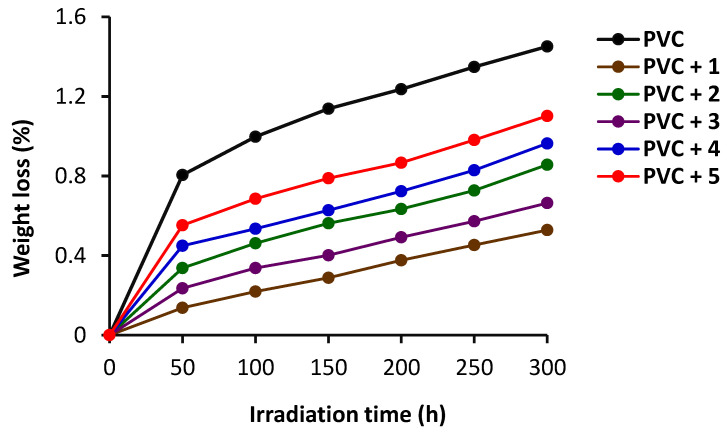
Weight loss (%) of the irradiated PVC films.

Photodegradation of PVC leads to the elimination of volatiles and causes a reduction in weight [56]. The weight loss percentages of the PVC films due to irradiation were calculated using Equation (2) and are presented graphically in Figure 7. Generally, weight loss increases as radiation time progresses, and it was sharpest in the first 100 h. The greatest weight loss was seen for the pure PVC film, and the use of methyldopa–tin complexes as photostabilizers led to a significant reduction in weight loss in comparison. At the end of irradiation, the weight loss (%) was 1.45% (blank PVC film), 0.53% (PVC + **1**), 0.86% (PVC + **2**), 0.66% (PVC + **3**), 0.96% (PVC + **4**), and 1.10% (PVC + **5**). Again, highly aromatic complexes (i.e., **1** and **3**) led to the highest stabilizing effect compared to the others.

### 3.5. Viscosity Average Molecular Weight (M_V_) Study of PVC Films

**Figure 8:**
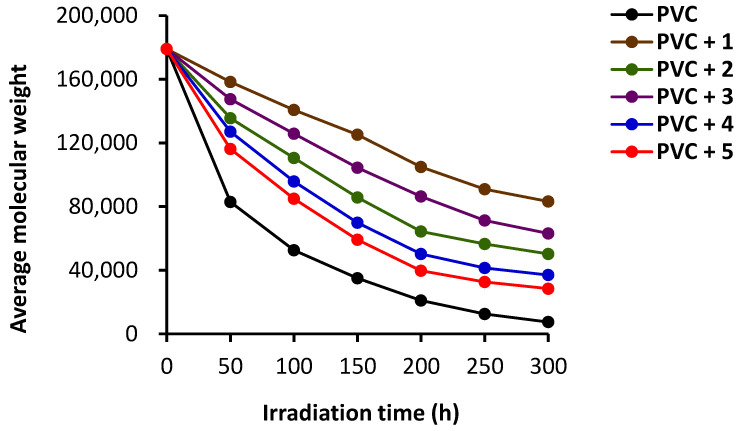
The Mv of the irradiated PVC films.

Both chain scission and cross-linking are common processes resulting from PVC photodegradation [54,57]. These processes can lead to a reduction in Mv due to the elimination of small fragments. The Mv for the irradiated PVC was calculated using Equation (3) for different irradiation times and is presented graphically in Figure 8. The viscosity of solutions of PVC dropped as irradiation time increased. The drop in Mv is generally massive and is initially particularly noticeable for pure PVC film. It was clear that the additives hindered the decrease in Mv. For pure PVC, the reduction in Mv was 54% after 50 h, 71% after 100 h, 88% after 200 h, and 96% after 300 h of irradiation. In contrast, the irradiated PVC film containing **1** led to a decrease in Mv by 12%, 21%, 41%, and 53% after 50, 100, 200, and 300 h of irradiation, respectively.

### 3.6. Surface Assessment of the Irradiated PVC Films

**Figure 9:**
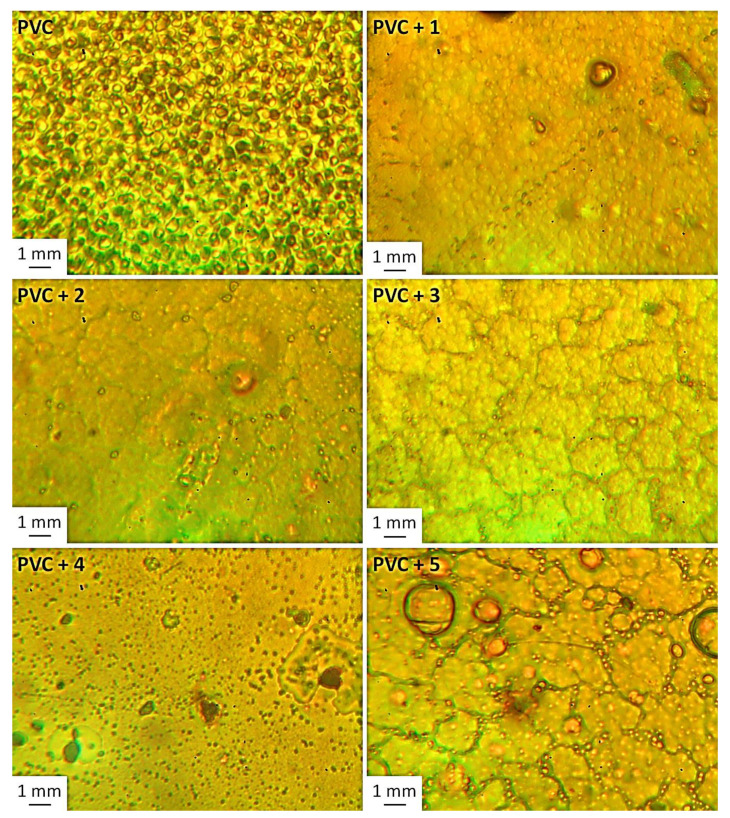
Images under the optical microscope for the irradiated PVC films.

**Figure 10:**
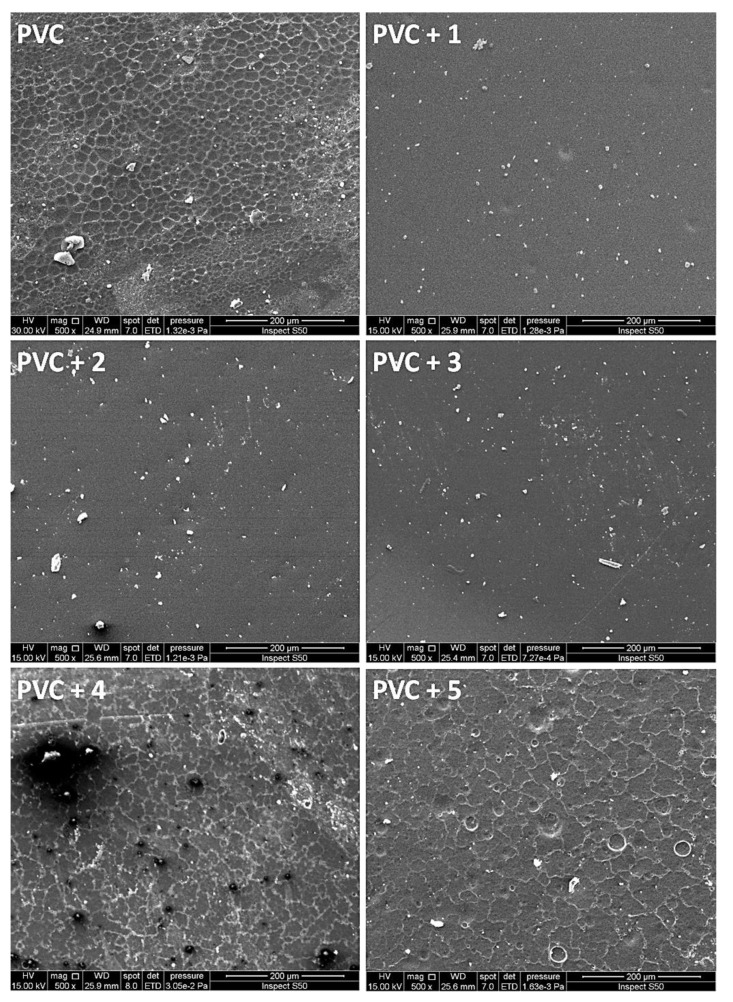
SEM images of the irradiated PVC films.

**Figure 11:**
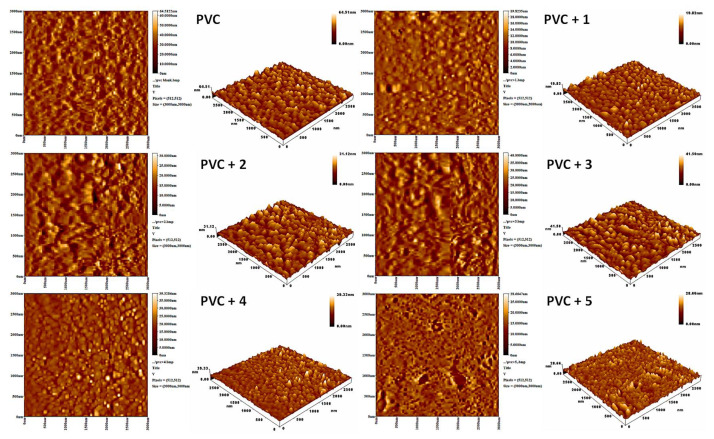
AFM images of the irradiated PVC films.

Various types of microscopy techniques, including optical, SEM, and AFM, have been utilized to assess the damage that occurs on the surface of irradiated PVC films [58,59,60,61]. These tools are good for monitoring the changes caused in the surface of polymers due to irradiation. In addition, the AFM provides clear non-distorted two- and three-dimensional images to provide a clear picture of the roughness factor of the irradiated surface. Generally, non-irradiated PVC films have a high degree of homogeneity, regularity, and smoothness [31]. Optical microscopy (Figure 9), SEM (Figure 10), and AFM (Figure 11) images revealed more damage (e.g., cracks, spots, darkness, irregularities, and roughness) on the surface of irradiated blank PVC in comparison to those containing **1**–**5**. For example, the SEM images indicated the random distribution and incorporation of metal complexes within the PVC blend. The elimination of HCl and volatiles, bond cleavages, and chain scission are the main causes of surface damage. The results provided additional evidence for the efficacy of methyldopa–tin complexes as PVC photostabilizers. The microscopic images of the surface of the irradiated PVC film containing complex **1** showed the least damage in terms of heterogeneity and roughness. Thus, for example, the roughness factor (Rq) for the irradiated films was 458.6 (blank PVC film), 28.3 (PVC + **1**), 52.8 (PVC + **2**), 40.2 (PVC + **3**), 68.3 (PVC + **4**), and 70.1 (PVC + **5**). It was clear that complex **1** led to a reduction in Rq by 16.2-fold, which is remarkable.

The use of methyldopa–tin complexes led to a significant improvement in the Rq and showed great efficiency in stabilizing PVC compared to many other PVC additives that have recently been reported [62,63,64,65,66,67,68,69,70,71,72]. For example, organotin complexes containing naproxen [62], carvedilol [63], furosemide [64], valsartan [65], telmisartan [66], trimethoprim [67], norfloxacin [68], and levofloxacin [69] led to a reduction in the Rq by 5.2–15.4 fold. On the other hand, organotin complexes with a high level of heteroatoms and aromaticity (e.g., ciprofloxacin, 4-(benzylideneamino)benzenesulfonamide, and 4-methoxybenzoic acid) resulted in greater improvement in the Rq (16.6–21.2 fold) [70,71,72].

### 3.7. Suggested Mechanisms for Photostabilization

**Scheme 3:**
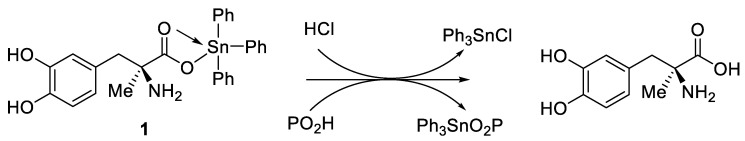
Complex **1** acts as an HCl and PO_2_H decomposer.

Methyldopa–tin complexes act as good PVC photostabilizers and reduce the damage due to photodegradation. The additives act as absorbers of harmful UV light, thereby protecting the polymer [73]. In addition, these stabilizers deactivate reactive species produced during photodegradation [74]. For example, the tin atom (a Lewis acid) in complex **1** can act as an acidic center and is, therefore, capable of scavenging the HCl (i.e., a secondary photostabilizer) released during photodegradation (Scheme 3). Moreover, the additives (e.g., complex **1**) act as decomposers for the hydroperoxides (PO_2_H; Scheme 3) produced by the photodegradation of PVC [75].

**Scheme 4:**
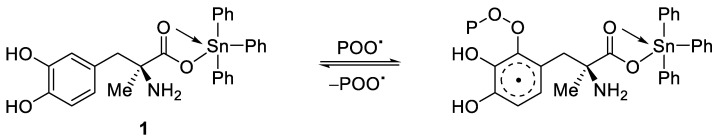
The function of complex **1** as a radical scavenger.

The reactive species (e.g., peroxide radicals, POO^•^) produced during PVC photodegradation can form stable intermediates with methyldopa–tin complexes (e.g., complex **1**) as a result of the resonance within the aryl ring [76]. Thus complex **1** acts as a radical scavenger and leads to a significant stabilization of the PVC (Scheme 4).

**Figure 12:**
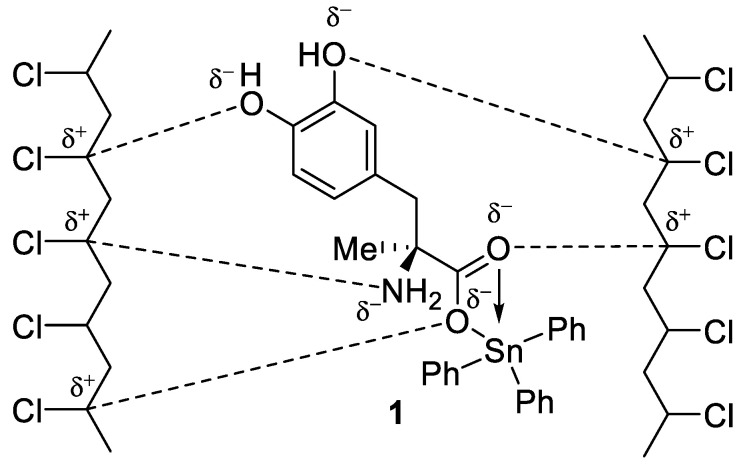
Coordination between PVC and complex **1** through polarized bonds.

Finally, the interaction between the polarized atoms within the PVC (i.e., C–Cl bonds) and the heteroatoms (oxygen and nitrogen) within the organic motif in complexes could stabilize the polymer (Figure 12). However, in large molecules, such as blended PVC, steric hindrance may interfere with the effectiveness of the process.

## 4. Conclusions

A simple procedure has been used to obtain, in high yield, five methyldopa–tin complexes containing aromatic and aliphatic substituents. Polyvinyl chloride thin films can be damaged by ultraviolet irradiation, and the complexes have been assessed as additives for stabilization against irradiation. The use of a small concentration of methyldopa–tin complexes as additives significantly improved the photostability of polyvinyl chloride. The methyldopa–tin complexes coordinate well with the polymeric chains and act by absorbing the radiation. In addition, they act as radical, peroxide, and hydrogen chloride scavengers. The most effective additives were the ones containing aromatic residues. The methyldopa–tin complexes clearly have the potential for application as stabilizers for polyvinyl chloride, but additional research is required to evaluate any health risks associated with these additives.

## Figures and Tables

**Table 1:** Some physical data for **1–5**.

Complex	Melting Point (°C)	Yield (%)	Calculated (Found; %)			
			C	H	N	Sn
**1**	105–107	83	60.03 (60.07)	4.86 (4.89)	2.50 (2.52)	21.19 (21.23)
**2**	174–176	74	52.82 (52.86)	7.86 (7.92)	2.80 (2.84)	23.73 (23.76)
**3**	123–125	84	55.43 (55.46)	9.94 (9.97)	4.04 (4.06)	17.12 (17.14)
**4**	108–110	81	51.47 (51.49)	6.48 (6.51)	4.29 (4.33)	18.17 (18.20)
**5**	178–180	78	46.42 (46.47)	5.31 (5.35)	4.92 (4.93)	20.86 (20.91)

**Table 2:** Selected FTIR spectral data for **1–5**.

Complex	NH_2_	OH	C=O Asym	C=O Sym	Δv (Asym―Sym)	Sn―C	Sn―O
**1**	3450, 3415	3271	1757	1487	270	511	443
**2**	3473, 3409	3217	1776	1491	285	530	455
**3**	3452, 3427	3277	1734	1491	243	522	443
**4**	3444, 3427	3252	1732	1493	239	524	443
**5**	3431	3279	1734	1497	237	514	457

**Table 3:** ^1^H NMR spectral data of **1–5**.

Complex	^1^H NMR (400 MHz)
**1**	8.91 (br s, 1H, OH), 8.79 (br s, 1H, OH), 7.92–7.66 (m, 15H, 3 Ph), 6.60 (s, 1H, Ar), 6.52 (d, *J* = 7 Hz, 1H, Ar), 6.29 (d, *J* = 7 Hz, 1H, Ar), 3.45 (br s, 2H, NH_2_), 2.90 (d, *J* = 14 Hz, 1H, 1H of CH_2_), 2.65 (d, *J* = 14 Hz, 1H, 1H of CH_2_) 1.28 (s, 3H, Me)
**2**	9.01 (br s, 1H, OH), 7.68 (br s, 1H, OH), 6.67 (d, *J* = 8 Hz, 1H, Ar), 6.64 (s, 1H, Ar), 6.51 (d, *J* = 8 Hz, 1H, Ar), 3.81 (br S, 2H, NH_2_), 2.92 (d, *J* = 13 Hz, 1H, 1H of CH_2_), 2.71 (d, *J* = 13 Hz, 1H, 1H of CH_2_), 1.64 (br s, 6H, 3 CH_2_), 1.56–1.48 (m, 12H, 6 CH_2_), 1.24 (s, 3H, Me), 0.85 (t, *J* = 7 Hz, 9H, 3 Me)
**3**	9.01 (br s, 2H, 2 OH), 8.90 (br s, 2H, 2 OH), 7.41–7.30 (m, 10H, 2 Ph), 6.68 (s, 2H, Ar), 6.62 (d, *J* = 8 Hz, 2H, Ar), 6.48 (d, *J* = 8 Hz, 2H, Ar), 3.73 (br s, 4H, 2 NH_2_), 2.92 (d, *J* = 14 Hz, 2H, 1H of CH_2_), 2.86 (d, *J* = 14 Hz, 2H, 1H of 2 CH_2_), 1.23 (s, 6H, 2 Me)
**4**	8.99 (br s, 2H, 2 OH), 8.12 (br s, 2H, 2 OH), 6.68 (d, *J* = 8 Hz, 2H, Ar), 6.63 (s, 2H, Ar), 6.50 (d, *J* = 8 Hz, 2H, Ar), 3.65 (br s, 4H, 2 NH_2_), 2.97 (d, *J* = 13 Hz, 2H, 1 H of 2 CH_2_), 2.81 (d, *J* = 13 Hz, 2H, 1H of 2 CH_2_), 1.66 (br s, 4H, 2 CH_2_), 1.51–1.42 (m, 8H, 4 CH_2_), 1.26 (s, 6H, 2 Me), 0.82 (t, *J* = 7 Hz, 6H, 2 Me).
**5**	88.88 (br s, 2H, 2 OH), 7.96 (br s, 2H, 2 OH), 6.65 (d, *J* = 8 Hz, 2H, Ar), 6.53 (s, 2H, Ar), 6.51 (d, *J* = 8 Hz, 2H, Ar), 3.74 (br s, 4H, 2 NH_2_), 2.96 (d, *J* = 14 Hz, 2H, 1 H of 2 CH_2_), 2.76 (d, *J* = 14 Hz, 2H, 1H of 2 CH_2_), 1.40 (s, 6H, 2 Me), 0.51 (s, 6H, 2 Me)

**Table 4:** ^13^C NMR spectral data of **1–5**.

Complex	^13^C NMR (100 MHz)
**1**	173.1, 145.4, 144.9, 136.7, 128.9, 128.8, 128.4, 125.6, 121.6, 118.6, 116.0, 60.7, 42.5, 23.1
**2**	173.5, 145.5, 145.0, 126.3, 121.7, 118.6, 116.1, 60.9, 42.6, 28.6, 27.0, 23.1, 20.9, 14.3
**3**	172.9, 145.6, 145.3, 136.9, 128.8, 128.7, 128.2, 124.8, 121.6, 118.2, 116.1, 60.2, 42.3, 22.3
**4**	173.0, 145.5, 145.2, 125.0, 121.6, 118.3, 116.1, 60.3, 42.3, 28.0, 26.1, 22.5, 22.2, 14.2
**5**	174.2, 145.5, 145.1, 125.5, 121.7, 118.5, 116.0, 60.6, 42.4, 23.0, 6.1

## Data Availability

Data are contained within the article.
